# Nitrogen nutrition in plants: rapid progress and new challenges

**DOI:** 10.1093/jxb/erx171

**Published:** 2017-06-27

**Authors:** Alain Gojon

**Affiliations:** UMR Biochimie & Physiologie Moléculaire des Plantes - (B&PMP), CNRS-INRA-SUPAGRO-UM, Campus INRA / SupAgro, Place Pierre Viala –, Montpellier Cedex, France

**Keywords:** Bio-geochemical nitrogen cycle, crop productivity, inorganic nitrogen, nitrate pollution, nitrogen nutrition, nitrogen sensing and signalling, nitrogen-use efficiency, transport

**As a main feature of plant autotrophy, assimilation of inorganic nitrogen is not only of fundamental scientific interest, but also a crucial factor in crop productivity. In its broad sense – from root uptake of various forms of N in the soil to allocation of N assimilates to different organs – it involves a wide range of physiological processes whose mechanisms are far from being fully understood. The aim of this special issue is to provide a wide overview of recent progress in this field, and to draw an interdisciplinary picture of the prospects for future research.**

Increasing demand for food, the requirement for a more environmentally friendly agriculture and future risks arising from climate change are all associated with the urgent need to improve N use efficiency in plants (Zhang *et al.*, 2015). For more than 50 years, N fertilizers have been an efficient way to enhance crop production. Further increases are an absolute requirement in meeting the needs of a rapidly growing population, but agriculture must now find alternative solutions to ensure adequate N nutrition of plants. Indeed, the amount of N fertilizers used worldwide is now so huge that it almost equals the natural global N fixation from the atmosphere into the bio-lithosphere. The consequence of such an enormous anthropogenic input is that the bio-geochemical N cycle is running out of control (Steffen *et al.*, 2015), resulting in major detrimental effects on the environment such as nitrate pollution of freshwater and coastal ecosystems (Galloway, 2003). Moreover, climate change brings an additional, unexpected threat as many studies highlight that the continuous elevation of atmospheric CO_2_ concentration will negatively impact the N status of most C_3_ plants, leading to lowered nutritional value of crops (Myers *et al.*, 2014).

Is there any hope of meeting this challenge successfully? The answer is certainly yes. In fact, it is now clear that the continuous decrease of N use efficiency in agriculture can be stopped and even reversed. Furthermore, increasing both yield and N use efficiency is possible and has already been achieved in several countries (Zhang *et al.*, 2015; Hawkesford, 2017). The problem is obviously multi-factorial, but plant scientists do have a pivotal role as the performance of individual crop genotypes is at the heart of the improvement of N use efficiency in agriculture. Innovative solutions aimed at ecological intensification will have to be proposed – for example, these might be based on beneficial interactions between plants and soil microorganisms, including N_2_-fixing symbioses. However, it is easy to predict that reducing the use of synthetic N fertilizers in agriculture will not be realistic for quite a while, and that a more efficient use of mineral N taken up from the soil will remain a major goal for plant breeding.

The ‘Nitrogen Nutrition of Plants’ symposia are milestones allowing the associated, interdisciplinary scientific community to exchange ideas on recent findings concerning the mechanisms of inorganic N assimilation by plants (Box 1). From the beginning, *Journal of Experimental Botany* has been at the leading edge in publishing research from this field (Box 2). The reviews in this special issue collectively represent a major update on this topic, covering a wide range of genetic, molecular, physiological and developmental aspects of N nutrition in various plant species. Furthermore, recent advances that will enable this fundamental research in model plants to improve N use efficiency in crops are considered.

**Box 1. Integration of nitrogen nutrition research**

The EMBO Conference Nitrogen2016 (Montpellier, France) continued a long-standing tradition of international conferences on nitrogen nutrition in plants, initiated in Europe by ENAAG (European Nitrate and Ammonium Assimilation Group, focusing on physiology and eco-physiology, 1986) and NAMGA (Nitrate Assimilation: Molecular and Genetic Aspects, 1982). Despite a common focus on the assimilation of inorganic nitrogen, these two groups initially functioned separately due to the different disciplines they represented, but as the borders separating them receded to give rise to new approaches (e.g. molecular physiology, functional genomics), the two organizations first held a joint meeting (Nitrogen2007, Lancaster, UK) and then merged. As a consequence, Nitrogen2007 was the founder meeting of a new series of conferences addressing a larger international audience, with the First (Nitrogen2010) and Second (Nitrogen2013) International Symposia on the Nitrogen Nutrition of Plants in Inuyama City (Japan, 2010) and Puerto Varas (Chile, 2013).

Although the initial focus on the assimilation of inorganic nitrogen is still strong, the Nitrogen Symposia now cover a wide range of approaches, allowing cross-fertilization of disciplines and fostering rapid progress in our understanding. Focusing on rice, the image summarizes current thinking on the contribution of NO_3_^−^ transporters for improving crop nitrogen use and efficiency (NUE) and yield (reproduced, with permission, from Fan *et al.*, 2017: yellow box, positive effects on rice NO_3_^−^ uptake, transport and remobilization in normal growth conditions; grey box, positive effects on NUE and yield under salinity; see Fan *et al.*, 2017, for further details).

**Figure:**
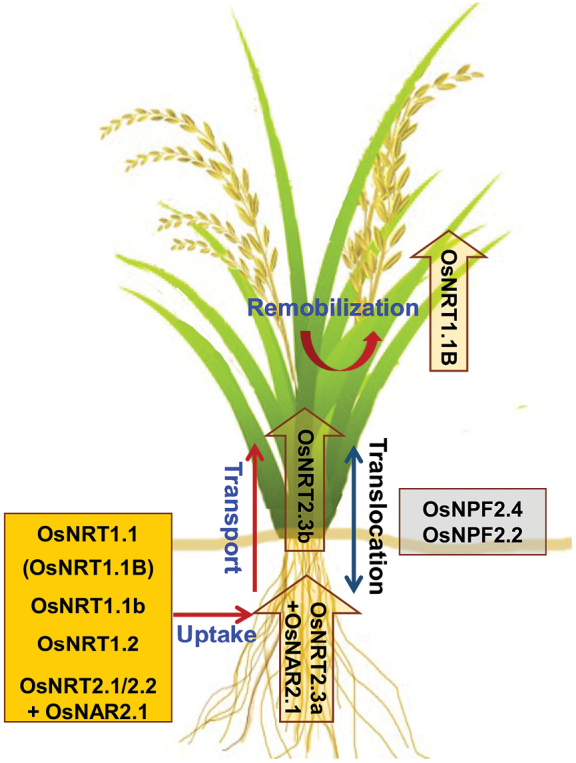

**Box 2. *Journal of Experimental Botany*: landmark publications on nitrogen nutrition**

*Journal of Experimental Botany* (*JXB*) has accompanied the Nitrogen Symposia from the very beginning, with dedicated special issues: ‘Nitrogen nutrition’ (Forde *et al.*, 2007); ‘Nitrogen: molecular biology, ecophysiology and beyond’ (Sakakibara *et al.*, 2011); and ‘Plant nitrogen nutrition’ in 2014 (see also reviews on nitrogen utilization in 2012: Saez *et al.*, 2012). Nitrogen nutrition is also prominent in related special issues, such as ‘Nutrient sensing and signalling’ (Takahashi, 2014) and ‘Plant membrane biology’ (Gilliham, 2011). Therefore, *JXB* is a landmark journal for those interested in a comprehensive and timely overview of current knowledge in the field. The image is taken from Hirel *et al.* (2007), one of the most highly cited *JXB* papers on nitrogen nutrition, appearing in the special issue associated with Nitrogen2007, the founder meeting of the current series of Nitrogen conferences. The maize ear phenotypes in GS1-deficient mutants and overexpressing lines are from N conditions which are suboptimal (N^+^) or limiting (N^–^): (A) wild type (WT), *gln1.4*, *gln1.3*, *gln1.3*/*gln1.4* mutants; (B) WT null segregants and T4 transgenic lines 1 and 9 overexpressing *Gln1-3* cDNA (reproduced, with permission, from Hirel *et al.*, 2007).

**Figure:**
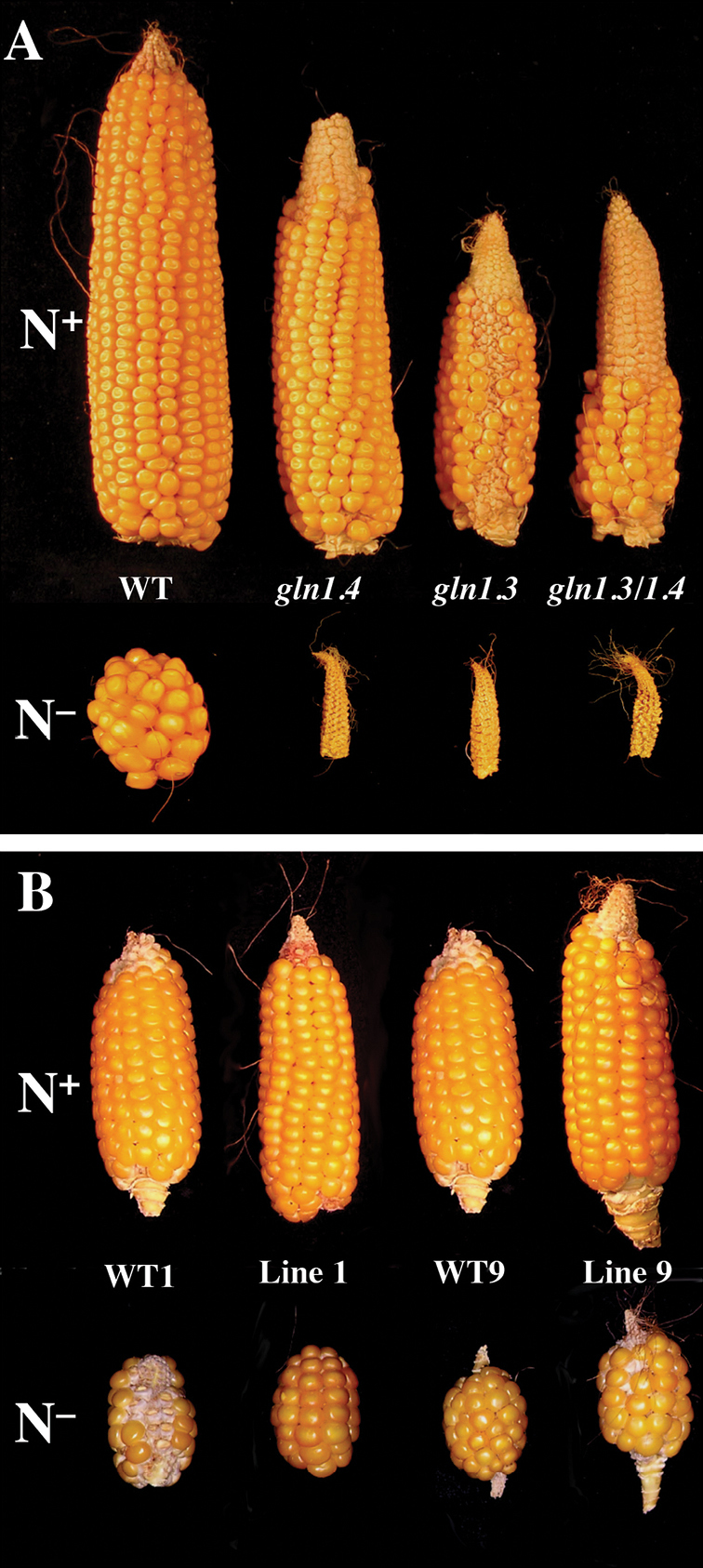

## Transport of nitrate and ammonium

Among the many different steps involved in inorganic N utilization by plants, those associated with the transport of nitrate and ammonium have received increasing attention over the past two decades. It is now clear that many (if not most) of the membrane transporter proteins involved have been identified at the molecular level in model species, and there is a general conservation of the related gene families between species, allowing fast progress in research on crops.

For nitrate, four gene families – *NPF* (*NRT1*), *NRT2*, *CLC* and *SLAC1/SLAH –* include all transporters and channels so far identified (Fan *et al.*, 2017; Li *et al.*, 2017). In Arabidopsis, these collectively represent more than 60 genes. Despite this complexity, nearly all steps of nitrate transport have been associated with the function of at least one member of these families: uptake by root cells, long-distance translocation between organs, and intracellular transport between cytoplasm and vacuole (Fan *et al.*, 2017). Molecular understanding of ammonium transport is focused on the AMT1 family of high-affinity transporters (Li *et al.*, 2017), which have a predominant role in root uptake. Further work is still needed to reach a full understanding of the mechanisms of nitrate and ammonium transport, but progress has already been impressive. For instance, all transporters contributing significantly to the high-affinity root uptake of these ions from the external medium have been identified in Arabidopsis, namely NRT2.1, NRT2.2, NRT2.4 and NRT2.5 for nitrate, and AMT1.1, AMT1.2, AMT1.3 and AMT1.5 for ammonium. This is evidenced by the phenotypes of multiple mutants in the corresponding genes, which are almost totally impaired in root N acquisition of either N source (Yuan *et al.*, 2007; Lezhneva *et al.*, 2014). Although this work of functional characterization is largely still to be completed in other species, the overall structure of the nitrate and ammonium transport systems seems to be rather well conserved across the plant kingdom (Fan *et al.*, 2017), and detailed genomic information is now available in many species, such as rice (Li *et al.*, 2017) and trees (Castro-Rodríguez *et al.*, 2017). However, marked specificities exist between different species, suggesting functional complexity that cannot be totally understood by just investigating model species like Arabidopsis. For instance, whereas the Arabidopsis AMT2 sub-family of high-affinity ammonium transporters is only represented by a single member of unclear function (AMT2.1), there are nine *AMT2* genes in the *Populus trichocarpa* genome (Castro-Rodriguez *et al.*, 2017).

When compared with this very active investigation of nitrate and ammonium transporters, elucidation of the molecular mechanisms of amino acid transport still appears to be at an early stage (Tegeder, 2012). However, transport of amino acids is essential for N partitioning between organs (Tegeder, 2014). Havé *et al.* (2017) confirm this in the context of N remobilization during senescence, a crucial process for seed N filling, stressing that little is known about the roles of many amino acid or peptide transporters up- or down-regulated during senescence.

## Nitrogen sensing and signalling: exciting times

Half of the reviews in this special issue deal with regulatory mechanisms involved in N sensing and signalling (Bellegarde *et al.*, 2017; Calatrava *et al.*, 2017; Forde and Gent, 2017; Hachiya and Sakakibara, 2017; Jacquot *et al.*, 2017; Liu and von Wirén, 2017; Undurraga *et al.*, 2017). This perfectly illustrates the current strong focus on these aspects, and rapid progress being made in our understanding. It has been known for a long time that inorganic N utilization by plants is finely regulated by highly complex and sophisticated signalling pathways, activated by sensing of both external N availability and internal N status of the whole plant (see Krapp *et al.*, 2014 and O’Brien *et al.*, 2016 for additional recent reviews).

Liu and von Wirén (2017) and Undurraga *et al.* (2017) document the role of both ammonium and nitrate as signalling molecules triggering a wide range of molecular, physiological and developmental responses. Findings concerning the sensing mechanisms of these two ions, and the associated downstream signalling events are coming out at an unprecedented pace. Interestingly, both reviews highlight the potential role of membrane transporters in ammonium or nitrate sensing, supporting the ‘transceptor’ (transporter/receptor) concept initially reported for the MEP2 ammonium transporter in yeast (Holsbeeks *et al.*, 2004). Indeed, increasing evidence indicates that the NPF6.3 (NRT1.1) nitrate transporter and the AMT1.1 and AMT1.3 ammonium transporters also serve as nitrate and ammonium sensors, respectively (see also Lima *et al.*, 2010; Gojon *et al.*, 2011; Bouguyon *et al.*, 2015). Although the downstream signalling pathways still remain obscure at the molecular level for ammonium, a significant number of regulatory genes or secondary signals have recently been reported for nitrate (Undurraga *et al.*, 2017), including NLP transcription factors (Marchive *et al.*, 2013) and calcium (Riveras *et al.*, 2015). In addition, Calatrava *et al.* (2017) point out the putative signalling role of nitric oxide (NO) in regulating nitrate transport and assimilation in *Chlamydomonas*. As studies on this model green alga have proved to be an important source of information for understanding processes of nitrate utilization in plants, it will be of interest to determine the general significance of this signalling role. Furthermore, ammonium and nitrate signalling are not independent from each other but strongly interact, as stressed by Hachiya and Sakakibara (2017), who review the many different effects of these interactions.

Besides perception of external availability of inorganic nitrogen through ammonium and nitrate sensing, plants also need to modulate N acquisition and utilization according to their N demand for growth. This relies on long-distance signalling of whole-plant N status, and associated local regulatory mechanisms. The mechanisms by which plants sense their N status are reviewed by Bellegarde *et al.* (2017) and Forde and Gent (2017). As highlighted by both reviews, and despite many interesting candidates, both sensors and long-distance signals of internal N status are unclear at the molecular level. However, and this is an additional illustration of the very active research in this area, new developments have occurred since the publication of these papers. Indeed, a very recent report indicates that long-distance signalling of nitrate availability is ensured by a root-to-shoot-to-root relay, involving xylem-mobile peptides and phloem-mobile glutaredoxin polypeptides (Ohkubo *et al.*, 2017). As is the case for nitrate signalling (Undurraga *et al.*, 2017), a significant number (>12) of regulators putatively acting downstream from the signals of N status have been identified in Arabidopsis (Bellegarde *et al.*, 2017), providing a first, albeit fragmentary view of the complex puzzle making up the control of N utilization by plants. However, most components listed in these two articles refer to regulators acting at the gene expression level, which is certainly only part of the story. Indeed, Jacquot *et al.* (2017) remind us that posttranslational regulatory mechanisms also play a major role, as evidenced by the data available for N transporters in both plants and microorganisms. For instance, phosphorylation is now known to be of major importance for controlling both transport and/or sensing functions of NPF6.3 (NRT1.1) and AMT1.1 in Arabidopsis (see also Liu and von Wirén, 2017).

Finally, although the elucidation of N sensing and signalling mechanisms now involves sophisticated functional genomics and systems biology approaches, much remains to be done at the phenotypic level to unravel all effects of N signalling in plants, and all responses of N acquisition and utilization to environmental factors. The reviews by Lin and Tsay (2017) and Bloom and Rubio-Asensio (2017) are both excellent illustrations of this statement. Indeed, although it has been suspected for a long time that N compounds could be signal molecules controlling flowering in plants, Lin and Tsay (2017) stands as a rare example of a putative comprehensive model for explaining the effect of N availability on flowering time in Arabidopsis. On the other hand, Bloom and Rubio-Asensio (2017) address the crucial question of the negative effect that the elevation of atmospheric CO_2_ concentration is predicted to have on the N status of most C_3_ plants. They document in detail, in Arabidopsis and wheat, the hypothesis that this effect is dependent on the N form provided to the plant, because of a specific inhibition of the assimilation of nitrate, but not of ammonium, by elevated CO_2_.

## From model plants to crops: rising concern

How can the fundamental knowledge acquired in model plants be used to speed up improvement of N use efficiency in crops? This is now a major question being addressed by many groups working on inorganic N utilization. Fan *et al.* (2017) and Li *et al.* (2017) review recent developments associated with this goal. As a consequence of our now detailed knowledge on the ammonium and nitrate transporter genes (see above), many attempts have been based on the manipulation of their expression, but there are only limited examples of success using this strategy to improve root N uptake in crops. The reasons for this are still unclear, but the selection of appropriate gene promoters may be crucially important in ensuring a positive outcome (Fan *et al.*, 2017; Li *et al.*, 2017); another reason may be related to the differences between species (Li *et al.*, 2017). Surprisingly, overexpression of nitrate transporter genes yielded better results in rice than in Arabidopsis, even though nitrate is not considered to be the preferred N source for rice. One of the best examples is the overexpression of the *OsNRT2.3b* gene in rice, which significantly enhanced growth, grain yield and N use efficiency, but through a mechanism more related to control of pH than direct stimulation of nitrate transport (Fan *et al.*, 2016). However, the hypothesis that ammonium or nitrate transporters are key players in determining N use efficiency is supported by quantitative genetic studies. This is exemplified by the finding that polymorphism in the *OsNRT1.1B* nitrate transporter gene is causal in the marked difference in N use efficiency between *japonica* and *indica* subspecies of rice (Hu *et al.*, 2015). Besides ammonium and nitrate transport, other processes of N utilization, such as N remobilization during senescence and protein accumulation in grains, or developmental traits such as root architecture, are also crucial for determining N use efficiency (Havé *et al.*, 2017; Hawkesford, 2017). Accordingly, several reports indicate that manipulation of organic N transport and allocation between organs is also a promising strategy for improving crop productivity (Tegeder, 2014).

## Future perspectives

Recent years have seen a tremendous increase in the number of genes found to be involved in the mechanisms of inorganic N utilization in plants, especially relating to signalling. Important pieces of the puzzle are still missing, such as regulators acting at the posttranslational level. However, based on our current knowledge there are at least four key pathways for future research.

First, we now need to understand how all these genes interact within the complex regulatory networks allowing plants to ensure adequate N nutrition under fluctuating environmental conditions. Working with so many genes at the same time will require high-throughput phenotyping methodologies for large-scale functional characterization studies, dedicated methods for investigating gene networks at the tissue-specific level, and mathematical modelling for understanding, and in the long term predicting, the functioning of these networks.

Second, there are now interesting parallels emerging between the regulatory mechanisms involved in the control of nitrate and/or ammonium acquisition and those involved in the control of symbiotic N_2_-fixation. For instance, NIN-like transcription factors are pivotal in the regulation of short-term responses to nitrate in Arabidopsis (Marchive *et al.*, 2013) and of nodulation in legumes (Schauser *et al.*, 2005). Also, a similar system of peptide signalling seems to be operating in the regulation of root nitrate uptake in Arabidopsis (Tabata *et al.*, 2014) and lateral root and nodule development in *Medicago* (Mohd-Radzman *et al.*, 2016). This clearly calls for more integrated studies of the various pathways of N acquisition in plants, and thus for more interactions between the two communities working on inorganic N assimilation and symbiotic N_2_ fixation.

Third, biotechnological attempts to manipulate candidate genes for improving N use efficiency in crops should be pursued, but using more refined methods (e.g. dedicated gene promoters, genome editing technologies, gene stacking). This is particularly important as our exploding knowledge about N signalling pathways is expected to provide an unprecedented number of new candidate genes. In fact, several examples confirm that altering the expression of regulators of N transporters (such as NAC or BT transcription factors) can lead to improved plant performance (Fan *et al.*, 2017; Li *et al.*, 2017). Furthermore, identifying beneficial alleles of key genes of N utilization is important. For selecting genotypes better adapted to low N input production systems, this will certainly require in-depth analysis of wild or ancient germplasms (Hawkesford, 2017).

Finally, scientists working on N nutrition of plants need to worry about climate change, and for at least two reasons. On the one hand, it is probably of strategic importance to understand why elevated atmospheric CO_2_ concentration is predicted to be so detrimental for the N status of most crops which are basic to human nutrition, with a reduction in protein content of edible parts that can be as high as 15–20% (Loladze, 2014; Myers *et al.*, 2014; Bloom and Rubio-Asensio, 2017). On the other hand, it also appears that N availability will be one major limiting factor preventing full stimulation of yield by elevated CO_2_ in C_3_ species (Reich *et al.*, 2006), yet more reason for improved N use efficiency in plants.
